# Long-term visual pathway alterations after elemental mercury poisoning: report of a series of 29 cases

**DOI:** 10.1186/s12995-021-00341-z

**Published:** 2021-11-12

**Authors:** Salvador Pastor-Idoate, Rosa M. Coco-Martin, Iratxe Zabalza, Yrbani Lantigua, Itziar Fernández, Jose L. Pérez-Castrillón, Ruben Cuadrado, Jose A. de Lazaro, Angela Morejon, Antonio Dueñas-Laita, Jose C. Pastor

**Affiliations:** 1 Institute of Applied Ophthalmobiology (IOBA), Eye Institute, University of Valladolid, Campus Miguel Delibes, P° de Belén n° 17, 47011 Valladolid, Spain; 2 Cooperative Health Network for Research in Ophthalmology Oftared, National Institute of Health Carlos III, ISCIII, Madrid, Spain; 3 Clinic University Hospital of Valladolid, Valladolid, Spain; 4 Puerta del Mar University Hospital of Cádiz, Cádiz, Spain; 5 University Hospital of Burgos, Burgos, Spain; 6 Department of Internal Medicine, Río Hortega University Hospital, University of Valladolid, Valladolid, Spain; 7 Unit of Clinical Toxicology, Río Hortega University Hospital, University of Valladolid, Valladolid, Spain

**Keywords:** Mercury poisoning, Mercury vapor, Occupational exposure, Optical coherence tomography (OCT), ocular Electrofisiology

## Abstract

**Background:**

There are few clinical data on retinal involvement after acute exposure to high concentrations mercury and the available reports are based on a small number of patients suffering chronic exposure. The purpose of this paper is to report findings in workers acutely exposed to very high concentrations of mercury vapor with the aim of providing data on a possible direct retinal involvement.

**Methods:**

Twenty-nine patients and 16 controls were evaluated in a comparative case series. Mercury levels in blood and urine samples, visual acuity (VA), contrast sensitivity (CS), visual field (VF), color discrimination and optical coherence tomography (OCT) were recorded. The pattern reversal visual-evoked potentials (PRVEP), full-field and multifocal electroretinography (ffERG/mfERG), pattern electroretinography (PERG), systemic symptoms, presence of erethism, and electromyography (EMG) were also gathered. A descriptive analysis was performed. The correlations between variables also were studied.

In addition, electrophysiological data from those patients with deeper VF defects (group 1) were compared with a normal control group.

**Results:**

Twenty-six workers exhibited symptoms of erethism. The EMG showed sensorimotor polyneuropathy and multiple mononeuropathy. The VA was slightly affected in 48.27% (*n* = 14) of subjects. Loss of CS in at least one of four spatial frequencies and color vision alterations occurred in 96.5% (*n* = 28) and 44.8% (*n* = 13), respectively. VF alterations were identified in 72.4% (*n* = 21) patients. No morphologic changes were seen in the OCT scans. Latencies over 100 milliseconds and reduced amplitudes of P100 were found in the PRVEP (*p* < 0.05). The reduced amplitude of the b wave at the ffERG, of the P50 at the PERG and of the P1 wave at the mfERG results (p < 0.05) suggested that the outer retina was involved. Significant negative correlations among blood mercury levels, VA, and ffERG were observed.

**Conclusions:**

In this case series, showed that acute exposure to mercury vapor had a hazardous effect on the visual system. Although neurologic and visual pathway involvement was clearly demonstrated, the differences found compared to control support the existence of a direct functional retinal damage and participation in impaired vision in mercury poisoning.

## Background

Episodes of acute or subacute poisonings as the result of exposure to elemental mercury in the workplace are fortunately uncommon. Toxic metals such as mercury have been implicated in several neurological disorders [1–4] and have been thought to be responsible for several retinal and optic disorders because of their proximity to the Central Nervous System (CNS) and the proved neurotoxicity of this metal [5–7]. However, it is still unclear whether the eye tissues lesions are consequence of CNS affectation or derived from a direct effect.

Furthermore, the location of the possible retinal involvement is unclear. Some animal studies have reported the accumulation of mercury in vitreous, retina and the choroid after systemic administration [8, 9], but others have limited the presence of the metal to the retinal pigment epithelium (RPE) and external neuroretinal layers [10–12] that is, to the structures irrigated by the choroid.

But the irruption, in clinic, of techniques that allow a detailed analysis of the function (electrophysiology or autofluorescence tests) and a detailed evaluation of the anatomy in a non-invasive and in vivo way by optical coherence tomography (OCT), enables clinicians with the possibility of contributing with important data to resolve this controversy.

The OCT provides important information about the normal or impaired structure of both the retina and optic nerve. Regarding electrophysiology, it is used to study the performance of the retina, optic nerve, and high visual pathway. It has multiple variants that allow a detailed analysis of many of the layers of the retina, e.g., the full-field electroretinogram (ffERG) gives an idea about the functioning of extensive areas of the retina; the multifocal electroretinogram (mfERG), assess early damage in small areas in retina, or the pattern electroretinogram (PERG) which provides information about macular area and retinal ganglion cell function [13, 14]. Finally, the visual evoked potentials (VEPs) offer important diagnostic information regarding the functional integrity of the visual system [13, 14].

As mentioned, physiologic and morphologic retinal changes resulting from mercury toxicity have been widely demonstrated in animal models [8–12] but there are few clinical reports showing clear effects on human retina from occupational poisoning which could have important implications in the valuation of handicaps and compensation; the last long series on mercury poisoning in humans were published before the latest retinal diagnostic techniques became available in clinic.

Only one group has reported OCT evaluation results, but on a group of patients who were chronically exposed [6, 15], and there is only one study on mfERG in patients chronically exposed to mercury showing color vision loss [16]. To the best of our knowledge, the current study is the first that includes a functional and structural study of the retina and optic nerve after acute mercury exposure.

The current study deals with one of the most severe incidents of acute elemental mercury intoxication in the European Union providing, unfortunately, the possibility of adding information on the controversial issue of direct retinal and optic nerve toxicity.

## Methods

This comparative case series followed the tenets of the Helsinki Declaration of 1964 (last amendment, 2013). The Clinical Research Ethics Committee of the Valladolid East Health Area approved the study and patients provided written informed consent.

### Patients

According to official company sources, 49 patients were exposed inadvertently to elemental mercury vapor while performing maintenance work in a heat exchanger. The incident occurred from November 19th to December 2nd, 2012, in a metal manufacturing plant in northern Spain. According to the workers’ stories, upon entering in the workspace, they observed balls of mercury spread over the floor*.* A few days after finishing their work, many of them presented with physical complaints that included asthenia, headache, lumbago, cough, bitter taste, dental pain, gum inflammation and bleeding, and epigastrium and abdominal pain among other symptoms, which were initially attributed to a viral infection.

After this initial symptomatology, most patients developed mercury-related erethism including fatigue, irritability, aggressiveness, anxiety, depression, and insomnia and neurologic manifestations that included tremor, peripheral polyneuropathy, weakness, headache, cognitive disorder, dizziness, and digestive manifestations such as diarrhea and abdominal cramps. Many of them also presented with visual complaints of blurred vision, ocular irritation, dry eye, burning or scratchy sensation, eye redness, and light sensitivity.

The levels of mercury in blood and urine, measured from the second week after the exposure, exceeded the biologic limits recommended for occupational exposure [17, 18], with some values between 500 and 900 μg/L in blood and between 600 and 1830 μg/g Cr in urine. Before the occupational exposure, the mercuric urinary levels measured in several of the affected workers, as a safety routine protocol, were below 3 μg/g Cr. However, no quantitative reference data were available about the level of mercury exposure at the time of the acute event.

Despite the range of early-stage symptoms, only three workers underwent early chelation with dimercaprol, also called British anti-Lewisite (BAL) which was interrupted prematurely by the appearance of severe adverse reactions related to this compound.

Between September 2013 and the end of 2014, 44 of the 49 affected patients presented to the Clinical Toxicology Unit of the Medical Science Institute of the University of Valladolid, Valladolid, Spain, for an independent assessment. After evaluation, different ancillary tests and actions were proposed based on individual patient’s clinical data. Twenty-nine of 44 subjects who presented with any visual symptoms were referred for a complete ophthalmologic evaluation at the Institute of Applied Ophthalmobiology (IOBA) Eye Institute of the University of Valladolid. Those without visual symptoms either at this time or in previous medical examinations were not considered for evaluation.

### Ophthalmic examination

At the beginning of the study, careful anamnesis was performed to rule out previous ocular, neural, or systemic diseases that could have affected the visual examinations.

Twenty-nine patients underwent a full ophthalmic examination that included measurement of intraocular pressure. Best-corrected visual acuity (BCVA) using the Early Treatment Diabetic Retinopathy Scale (ETDRS), slit-lamp examination, funduscopy, and OCT, with particular attention to evaluation of the central retinal thickness (CRT) (3D-OCT 2000, Topcon Inc., Tokyo, Japan) and retinal nerve fiber layer thickness (RNFLT) (OCT Stratus 3000 Zeiss Meditec, Oberkochen, Germany). Color vision was evaluated by using the Roth 28 Hue Test (Lunean Ophtalmologie, Paris, France) and contrast sensitivity (CS) by using the CSV-1000 chart (Vectorvision, Greenville, OH). The results of the color vision assessment were scored in two ways. First, a color confusion index (CCI) was calculated for each participant for statistical analysis [19–21]. Second, a clinical diagnosis of the type of loss was stablished by plotting responses on a standard score sheet. This allowed the determination of the axis of color confusion. Based on the major confusion axis, a diagnosis of normal, red-green (protan), blue- yellow (tritan), mixed, or non-specific deficiency could be established.

Visual fields (VFs) were assessed using the Humphrey 750i Visual Field Analyzer (Carl Zeiss, Oberkochen, Germany) and the central 30–2 SITA fast strategy protocol. Only tests that met the criteria [low (< 20%), false positive, false negative, and fixation loss parameters] were considered.

Pattern reversal visual-evoked potentials (PRVEP) and ERG recordings were assessed using a computerized Optoelectronic Stimulator Vision Monitor MonPack 120 Metrovision (Pérenchies, France) according to the International Society for Clinical Electrophysiology of Vision (ISCEV) protocols [13, 14]. ffERGs, PERGs, and mfERGs from both eyes were recorded of each patient. Four patterns of abnormal mfERG amplitude responses were assessed: paracentral loss, foveal loss, peripheral loss, and generalized loss, as described by Maturi et al. [22].

Following the ISCEV protocols it is possible to discern between rod function (scotopic responses) or cone function (photopic responses); as well as differentiate between damage at the outer (a wave) or inner retina (b wave and oscillatory potentials -OP-) [13, 14].

### Additional tests

Peripheral neuropathy was assessed by electromyography (EMG) using standard protocol and a computerized system (Nihon Kodhen, Model MEB-9400, Irvine, CA). Sensory and motor nerve conduction velocities were determined in the median and peroneal nerves. Amplitude (μV), latency (m/s), and conductance (m/s) were evaluated.

### Statistical analysis

Statistical analyses were performed using SPSS statistics 17.0 (SPSS, Inc., Chicago, IL). The BCVA was recorded using the ETDRS scale and converted to the logarithm of minimal angle of resolution (logMAR) for statistical analysis. All VA results are expressed in logMAR units with Snellen equivalent in parenthesis. Categorical variables were analyzed using Fisher’s exact test or chi-square test. The t-test was used to compare the mean values of the parametric values. Pearson’s correlation test was used to evaluate the correlation between ophthalmic findings and mercury levels in the blood and urine. For data without normal distribution, continuous variables were analyzed using the Wilcoxon rank-sum test. For repeated measures, the Wilcoxon signed-rank test was used, and Spearman test was performed for the correlation non-normally distributed data. For all tests, *P* < 0.05 was considered significant.

For statistical analyses, normative databases of OCT metrics for RNFLT and CRT were used.

Scotomas (blind areas in visual fields) do not always correlate with other visual functional tests. That means, in patients with enough preserved visual acuity and a normal-appearing visual field test, mfERG results can be abnormal [23, 24]. Thus, we decided, for the electrophysiology assessment, to separately analyze a sub-group (group 1) of patients who had deeper and more extensive defects in the VF tests. This may be particularly useful for a better characterization of the affected cell types and retinal layers.

In addition, a healthy group (*n* = 16) was used as control. Healthy individuals’ group with normal ophthalmologic evaluations is a fundamental requirement in functional tests since data from the literature cannot be used as a reference and normal values adapted to the specific clinical setting are required.

## Results

All 29 patients were men (mean age, 40.62 ± 8.05, range, 25–56). The mean urinary mercury concentration closer to the event was 302.86 ± 405.36 μg/g Cr; range, 10–1830); the mean blood mercury concentration was 392.93 ± 273.85 μg/L (range, 26–961). The main clinical baseline characteristics and EMG results are summarized in Table. 1. As mentioned, an age-matched healthy group (*n* = 16) (age, 43.44 ± 8.30 years, *p* = 0.271) was included for electrophysiological comparisons.

**Table 1:** Baseline characteristics of participants, laboratory, and electromyography findings

		*Patients* *n* = 29	*Controls* *n* = 16	*p-value*
Age, yrs. (mean, SD)		40.62 (8.05)	43.44 (8.30)	0.271
Smoking		18/29 (62.1%)	2/16 (12.5%)	0.001
Hypertension		2/29 (6.9%)	1/16 (6.25%)	> 0.05
Dyslipidemia		4/29 (13.8%)	2/16 (12.5%)	> 0.05
Psychiatric treatment		13/29 (44.8%)	–	–
Erethism		26/29 (89.7%)	–	–
Laboratory	Blood Hg (μg/L) (mean, SD)	392.93 (273.85)	–	–
	Urine Hg (μg/g Cr) (mean, SD)	302.86 (405.36)	–	–
EMG patterns	Normal	1 (3.4%)	–	-
	SP	14 (48.3%)	–	
	ASP	7 (24.1%)	–	
	MM	4 (13.8%)	–	
	N/A	3 (10.3%)	–	
EMG CVA	SN (ms) (mean, SD)	33.5 (7.13)	^a^ > 40	–
	MN (ms) (mean, SD)	38.78 (6.65)	^a^ > 49	

Data are presented as mean (SD) or as numbers

*Yrs*. years, *EMG* electromyography, *μg/L* microgram/liter, *μg/g* Cr microgram per gram of creatinine, *SP* sensorimotor polyneuropathy, *ASP* axonal sensory polyneuropathy, *MM* multiple mononeuropathy, *N/A* not performed. *EMG CVA* Electromyography = conduction velocity assessment, *SN* Sensory nerve, *MN* Motor nerve, *ms* milliseconds

^a^Reference values (Stetson 1992; Sedano 2013) = normal velocity conduction in SN > 40 milliseconds. Normal velocity conduction in MN > 49 milliseconds

### Ophthalmologic findings

The main ophthalmic findings are shown in Table. 2. The VA decreased (< 20/20) in fourteen patients (48.27%). The mean BCVA LogMAR was 0.048 ± 0.126. In addition, 15 (51.7%) of 29 patients presented with additional unspecific ocular complaints such as dry eye or eye redness and light sensitivity.

**Table 2:** Ophthalmic Examination Findings

		*Patients* *n* = 29		*Normal reference data*
N (eyes)		29 (58)		–
BCVA Logmar (mean, SD)		0.048 (0.126)		0.0
[Snellen] (mean, SD)		[0.920 (0.205)]		[6/6]
CVS		13 (44.8%)		–
Color patterns	Normal	16 (55.2%)		–
	Red-green defect	2 (6.8%)		
	Blue-yellow defect	9 (31.03%)		
	Mixed	1(3.44%)		
	Non-specific deficiency	1(3.44%)		
CCI (mean, SD)		1.642 (1.183)		1.0
CSA	Eye (RE, LE)	RE	LE	–
	CS3 mean (SD)	5.93 (1.22)	5.69 (1.16)	–
	CS6 mean (SD)	5.62 (1.08)	5.69 (1.31)	–
	CS12 mean (SD)	3.37 (1.01)	3.48 (0.91)	–
	CS18 mean (SD)	3.34 (1.34)	3.41 (1.52)	–
VF	Eye (RE, LE)	RE	LE	–
	MD mean (SD)	−5.64 (7.92)	−6.87 (8.52)	0.0
	VFI mean (SD)	86.7 (21.2)	85.4 (21.4)	100%
	Total, patients with alterations	21 (72.4%)		
OCT	Eye (RE, LE)	RE	LE	
	CRT mean (SD)	249.4 (21,0)	248.1 (20,7)	233.6 (19.7)
	RNFLT mean (SD),	102.2 (10.5)	100.2 (11.3)	100 (18)

Data are presented as mean (SD) or as numbers. *BCVA* best-corrected visual acuity, *RE* right eye, *LE* left eye, *CVS* color vision scores, *CCI* color confusion index, *CSA* alterations in the achromatic contrast sensitivity, *CS*3 spatial frequency at 3 cycles/degree, *CS*6 spatial frequency at 6 cycles/degree, *CS*12 spatial frequency at 12 cycles/degree, *CS*18 spatial frequency at 18 cycles/degree, *VF* visual field test, *MD* mean deviation, *VFI* Visual Field Index, *OCT* optical coherence tomography, *CRT* central retinal thickness, *RNFLT* retinal nerve fiber layer thickness

Acquired alteration in color vision, mainly in the blue-yellow range, occurred in 13 (44.8%) patients. The mean CCI was 1.642 ± 1.183 (normal value is 1.0 and higher values indicate poorer color discrimination) [19–21].

Twenty-eight (96.5%) patients showed changes in the achromatic CS in at least one of the four spatial frequencies and 21 (72.4%) patients had VF alterations (Table. 2). The most prevalent patterns were concentric constriction (17 eyes, 29.3%), scattered defects (6 eyes, 10.3%), hemifield defects respecting the horizontal and vertical meridians (5 eyes, 8.6%), nasal defects (4 eyes, 6.9%), and arcuate defects (2 eyes, 3.4%).

OCT did not show significant differences when the CRT and RNFLT measurements were compared to values in the normative SD-OCT databases [25, 26] (Table. 2).

Correlation analyses between the blood mercury levels (BML) and all above variables showed only a significant negative correlation with the BCVA (r = − 0.36, *p* = 0.048).

### Electrophysiology function assessment

#### ffERGs

The ffERGs were recorded in 28 patients and 16 controls. Statistical differences were found when comparing all patients’ values with those obtained from normal subjects, being lower the b-wave amplitude in SRR, the a-wave amplitude in MSR, and the sum of oscillatory potential (OPs) in cases than in controls (Table. 3).

**Table 3:** Full-Field ERGs. Amplitude of a- and b-Waves for the SRR, MSR, OP, Flicker 30 Hz and SFCR

Full-field ERGs			All patients	Control group	*p-v*alues	95%CI	Group 1^a^	Control group	*p-values*	95%CI
SRR	a-wave (μV)	RE	−14.74 ± 18.6	− 11.39 ± 13.56	0.5322	–	− 10.25 ± 4.23	−11.39 ± 13.56	0.7923	–
		LE	−8.0 ± 9.06	− 6.81 ± 7.36	0.6595	–	− 9.90 ± 4.53	−6.81 ± 7.36	0.2278	–
	b-wave (μV)	RE	264.42 ± 76.3	210.6 ± 80.49	**0.0329**	[− 103,0 to − 4,6]	188.0 ± 55.09	210.6 ± 80.49	0.4268	–
		LE	262.03 ± 76.7	203.3 ± 77.83	**0.0194**	[−107,5 to − 9,9]	182.3 ± 49.46	203.3 ± 77.83	0.4373	–
MSR	a-wave (μV)	RE	−160.21 ± 58.8	− 196.9 ± 55.01	**0.0480**	[−73,0 to − 0,33]	−103.7 ± 31.6	−196.9 ± 55.01	**< 0.0001**	[−131.2 to − 55.2]
		LE	−159.37 ± 63.6	− 187.2 ± 48.16	0.1369	–	− 102.1 ± 28.1	− 187.2 ± 48.16	**< 0.0001**	[− 118.4, to − 51.7]
	b-wave (μV)	RE	414.67 ± 86.4	413.3 ± 102,7	0.9626	–	331.3 ± 82.24	413.3 ± 102,7	**0.037**	[53.29 to 158.7]
		LE	403.92 ± 92.6	421.8 ± 96.90	0.5478	–	319.3 ± 83.02	421.8 ± 96.90	**0.0085**	[28.61, to 176.4]
OP	amplitude (μV)	RE	235.9 ± 106.5	570.02 ± 254.9	**< 0.0001**	[198,7 to 469,5]	309.5 ± 71.32	570.02 ± 254.9	**0.003**	–
		LE	252.5 ± 175.8	525.93 ± 239.4	**0.0003**	[135,0 to 411,8]	292.0 ± 73.8	525.93 ± 239.4	**0.004**	–
Flicker 30 Hz	b-wave (μV)	RE	90.40 ± 28.5	85.46 ± 19.17	0.5408	–	64.75 ± 9.87	85.46 ± 19.17	**0.0030**	[7.716 to 33.70]
		LE	90.13 ± 32.8	80.69 ± 17.49	0.2798	–	61.02 ± 7.56	80.69 ± 17.49	**0.0018**	[8.081 to 31.26]
SFCR	a-wave (μV)	RE	−13.19 ± 5.72	− 13.30 ± 13.43	0.9699	–	−9.85 ± 2.38	−13.30 ± 13.43	0.4100	–
		LE	−13.62 ± 6.15	− 14.28 ± 14.72	0.8356	–	− 9.92 ± 3.55	− 14.28 ± 14.72	0.3473	–
	b-wave (μV)	RE	65.09 ± 22.6	91.08 ± 11.5	0.2555	–	44.50 ± 14.08	91.08 ± 11.5	**< 0.0001**	[36.42 to 56.74]
		LE	64.04 ± 24.7	56,89 ± 15.07	0.3003	–	44.65 ± 14.65	56,89 ± 15.07	**0.0463**	[0.217 to 24.2]

Data are presented as mean ± SD or as numbers. *SRR* scotopic rod response, *MSR* maximal scotopic response, *OP* oscillatory potential, *Flicker 30 Hz* flicker 30 Hz, *SFCR* single flash cone response, *μV* microvolts; *RE* right eye, *LE* left eye, *CI* confidence interval, *Amp* amplitude

^a^Group 1 (*n* = 11): constituted by a subgroup of patients with evident visual disturbances in their VF. Patients with concentric constriction (17 eyes) and hemi-field defects (5 eyes) patterns

Since about half of the patients had not a clinically detectable visual impairment, we focused our attention on the results obtained from patients with relevant VF defects (G1) These data showed lower amplitudes when compared to control in all ffERG parameters, being these differences significant for MSR and 30-Hz flicker for the a- and b-waves and for the b-wave in the SFCR and OPs (Table. 3). These results were consistent with those reported previously by Ventura et al. 2004 [16] in mercury intoxicated patients.

No significant correlation was seen between the BML and ffERG parameters in the whole group of patients.

#### PERG

PERG was performed in 27 right eyes and in 26 left eyes of patients and in 14 controls (both eyes) (Table. 4). Despite showing reduced amplitudes in P50 and N95, there were no significant differences between patients and control group. However, this trend seen in all patients became significant when patients with impaired VFs (G1) were compared with control (Table. 4), in accordance with previous studies [27].

**Table 4:** PERG in Patients and Control Group

PERG			All patients	Control values	*p-values*	Group 1^a^	Control values	*p-values*	95%CI
P50	Amplitude (μV)	RE	4.32 ± 1.51	4.92 ± 1.66	4.92 ± 1.66	3.13 ± 0.67	4.92 ± 1.66	**0.0014**	[0.76 to 2.81]
		LE	4.35 ± 1.78	4.58 ± 0.91	4.58 ± 0.91	2.93 ± 0.81	4.58 ± 0.91	**0.0232**	[0.21 to 2.61]
N95	Amplitude (μV)	RE	−5.90 ± .293	− 6.65 ± 2.06	−6.65 ± 2.06	−4.00 ± 0.84	−6.65 ± 2.06	**0.0006**	[−4.01 to 1.27]
		LE	−5.19 ± .460	−5.65 ± 1.63	−5.65 ± 1.63	−3.19 ± 0.94	−5.65 ± 1.63	**0.0002**	[−3.60 to 1.31]

Data are presented as mean ± SD or as numbers. *Amp* amplitude, *PERG* pattern electroretinogram, *CI* confidence interval, μV microvolts, *RE* right eye, *LE* left eye

^a^Group 1 is a subgroup of patients with evident visual disturbances in their visual field test. Patients with concentric constriction (17 eyes) and hemi-field defects (5 eyes) patterns

There was no correlation between the PERG values and the BML.

#### PRVEP

PRVEP was recorded in 29 workers and 14 controls. The average implicit times of P100 and amplitudes did not differ between patients and controls for the 60- and 30-min checkerboard stimuli except in the implicit times of P100 for both 60 and 30 for the left eyes; but significant differences were seen when G1 was compared to control (Table. 5). However, no correlation was seen between PRVEP values and BML.

**Table 5:** PRVER in Patients and Control Group

PRVEP			All patients	Control values	*p-values*	95% CI	Group 1^a^	Control values	*p-values*	95% CI
P100-Da 60 R Lob	Amplitude (μV)	RE	8.82 ± 4.33	7.29 ± 1.59	0.2852	–	5.05 ± 1.03	7.29 ± 1.59	**0.0011**	[1.020 to 3.450]
		LE	8.45 ± 4.36		0.4193	–				
	Latency (ms)	RE	115.17 ± 10.21	112.6 ± 7.19	0.4684	–	130.9 ± 8.17	112.6 ± 7.19	**< 0.0001**	[−25,36 to − 11,24]
		LE	118.06 ± 9.71		0.1127	–				
P100-Da 60 L Lob	Amplitude (μV)	RE	8.88 ± 7.44	6.33 ± 2.40	0.2974	–	5.15 ± 1.02	6.33 ± 2.40	0.1538	–
		LE	8 ± 3.65		0.1872	–				
	Latency (ms)	RE	114 ± 9.24	111.2 ± 4.80	0.3681	–	132.0 ± 7.09	111.2 ± 4.80	**< 0.0001**	[−26,39 to − 15,21]
		LE	118.47 ± 10.36		**0.0420**	[−14,1 to −0,27]				
P100-Da 30 R Lob	Amplitude (μV)	RE	8.29 ± 3.72	6.29 ± 2.16	0.1179	–	4.40 ± 1.56	6.29 ± 2.16	**0.0319**	[0,1813 to 3,59]
		LE	7.44 ± 3.91		0.3846	–				
	Latency (ms)	RE	116.79 ± 6.21	112.12 ± 8.02	0.0588	–	134.7 ± 15.6	112.12 ± 8.02	**0.0006**	[−34,03 to −10,97]
		LE	122.38 ± 13.51		**0.0283**	[−19,5 to − 1,16]				
P100-Da 30 L Lob	Amplitude (μV)	RE	7.94 ± 4.01	5.93 ± 2.01	0.1392	–	3.85 ± 1.44	5.93 ± 2.01	**0.0128**	[0,494 to 3,66]
		LE	6.48 ± 3.78		0.6649	–				
	Latency (ms)	RE	117.14 ± 7.45	112.4 ± 7.32	0.0898	–	136.8 ± 22.4	112.4 ± 7.32	**0.0039**	[−39,96 to −8,84]
		LE	122.93 ± 17.36		0.0724	–				

Data are presented as mean ± SD or as numbers. *PRVF* pattern reversal visual evoked potential, *P100-Da 60* P100 wave with 60′ checkerboard stimuli, *P100-Da 30* P100 wave with 30′ checkerboard stimuli, *R Lob* right occipital cortex, *L Lob* left occipital cortex, *μV* microvolts, *RE* right eye, *LE* left eye, *CI* confidence interval

^a^Group 1 is a subgroup of patients with evident visual disturbances in their visual field test. Patients with concentric constriction (17 eyes) and hemi-field defects (5 eyes) patterns

#### mfERG

The mfERGs were recorded in 26 of 29 patients and 11 controls. The most prevalent patterns were peripheral loss (16 eyes, 30.7%) and central loss (8 eyes, 15.4%), followed by paracentral defects (6 eyes, 11.5%). Normal amplitude responses were observed in 22 eyes (42.3%). Because the peripheral pattern was the most frequently found, the N1/P1 amplitude ratio in the peripheral rings of the mfERG was analyzed; a significantly lower value was seen in patients in rings 5^o^ to 10° and > 15° compared to control (Table. 6). Additional significant differences at rings 1, < 2°, ring 2, 2^o^ to 5°, ring 3, 5° to 10°, ring 5, and > 15° were found when G1 was compared to controls (Table. 6). These results are consistent with previous studies [22, 28]. No correlations were seen between mfERG and BML values.

**Table 6:** mfERG Values in Patients and Control Group

Amplitude P1/N1ratio	**Ring**	**Eye**	**All patients**	**Control** **values**	*p-value*	**95%CI**	**Group 1**^a^	**Control** **values**	*p-value*	**95%CI**
	Ring 1	RE	380 ± 209.6	528 ± 104,7	**0.0334**	[12.35 to 283.7]	298.6 ± 137.4	528 ± 104,7	**0.0003**	[120.8 to 338.0]
		LE	278.4 ± 100.1		**< 0.0001**	[175.5 to 323.7]	182.0 ± 64.13		**< 0.0001**	[268.8 to 423.2]
	Ring 2	RE	124 ± 39.30	122.8 ± 12.69	0.9222	–	89.99 ± 29.32	122.8 ± 12.69	**0.0028**	[12.72 to 52.90]
		LE	123.2 ± 42.59		0.9760	–	81.45 ± 27.35		**0.0002**	[22.39 to 60.31]
	Ring 3	RE	42.35 ± 10.95	56.47 ± 6.81	0.3031	–	32.55 ± 7.470	56.47 ± 6.81	**< 0.0001**	[18.56 to 31.28]
		LE	43.74 ± 10.10		0.3503	–	34.43 ± 6.273		**< 0.0001**	[17.21 to 28.87]
	Ring 4	RE	43.01 ± 12.77	35.85 ± 4.72	0.4736	–	31.11 ± 8.031	35.85 ± 4.72	0.1070	–
		LE	43.06 ± 11.62		0.4646	–	32.08 ± 8.246		0.2031	–
	Ring 5	RE	12.36 ± 3.20	19.76 ± 1.21	**< 0.0001**	[5.370 to 9.430]	9.479 ± 1.489	19.76 ± 1.21	**< 0.0001**	[9.074 to 11.49]
		LE	12.5 ± 3.0		**< 0.0001**	[5.349 to 9.171]	9.802 ± 1.929		**< 0.0001**	[8.526 to 11.39]

Data are presented as mean ± SD or as numbers. Amplitude P1/N1 ratio (nV/deg^2^). Ring 1 = < 2°; ring 2 = 2–5°; ring 3 = 5°–10°; ring 4 = 10°–15°; and ring 5 = > 15, *RE* right eye, *LE* left eye, *CI* confidence interval

^a^Group 1 is a subgroup of patients with evident visual disturbances in their visual field test. Patients with concentric constriction (17 eyes) and hemi-field defects (5 eyes) patterns

Although comparability between mfERG and perimetry is limited, we tried to assess the VF defects patterns with the mfERG dysfunction patterns obtained in the three-dimensional plots. These patterns were subjectively assessed based on comparative methods and approaches previously used [29, 30]. The data showed different patterns between the mfERG defects and the total deviation of sensitivities in their VF tests in 14 (48.3%) patients. Eight (27.5%) patients had similar peripheral pattern defects in both tests, and four (13.8%) patients had mixed patterns; in three (10.3%) cases was no possible to establish any correlation.

### Additional tests

#### EMG

EMG, performed in 27 of 29 workers, showed different abnormality patterns and decreased nerve conduction velocity in most of them (Table. 1). No correlation was seen between BML, nerve conduction velocity, and the P100 component in PRVEP in the whole sample.

## Discussion

As mentioned, this study was conducted to evaluate morphological changes in retinal anatomy as assessed by OCT or in retinal cell function as assessed by various forms of ERG as well as its correlation with BM. They are procedures widely used in human clinics and data from these two methods constitute an important contribution to resolve controversies in retinal participation in this intoxication.

Mercury vapor is a significant source of mercuric load in occupational exposure because it is odorless and colorless and tends to accumulate in poorly ventilated areas. Once the lungs have absorbed the inhaled vapor, the mercury can reach different tissues via the bloodstream, with the primary target of the CNS and the eye [2, 31]. When it is oxidized, it cannot penetrate the blood-barrier again and remains for prolonged periods of time in tissues [2, 6, 7, 15, 31].

As mentioned, the neurologic and thus the visual pathway effects resulting from mercury toxicity have been described widely [2, 31, 32]. The long-term exposure neurological effects can include symptoms from tremor, neuropathy, personality changes referred to as mercurial erethism, speech disruption, delirium, or rigidity to symptoms of VF defects, reduced VA, color and night vision disfunction, or decreased CS [2, 7, 31, 33]. However, after the introduction of electrophysiology there is a strong suspicion that the retina may also be primarily affected and that not all alterations of the visual pathway are due to CNS poisoning [16].

As previously mentioned, in this event the first patients’ complaints were attributed to a viral infection, which delayed the diagnosis and analytical determinations. At the time of correct diagnosis, the mercuric values in urine (mean, 302.86 μg/g Cr) and blood (mean, 392.93 μg/L) significantly exceeded the maximal accepted level for occupational exposure (< 30 μg/g Cr and 10 μg/L, respectively) [17, 18]. In such cases, the mainstay of treatment is chelation therapy; however, only three patients underwent early chelation, which was stopped prematurely because of severe adverse reactions. Fifteen workers underwent delayed chelation (8 to 12 months after the initial incident). However, this late chelation did not result in significant symptom relief.

Twenty-six workers exhibited symptoms related to erethism. Some, also showed symptoms associated with cognitive mercury poisoning such as memory and attention disturbances [31, 32]. Tremor of the hands, head, and eyelids, a late symptom of mercury poisoning, also occurred in some patients. EMG showed signs of mixed sensorimotor polyneuropathy and multiple mono-neuropathy alterations 12 to 18 months after exposure.

In this series, the VA slightly decreased in fourteen patients; however, advanced visual functions were impaired apparently in an independent way from mercury levels since significant negative correlations were detected only among the BML, BCVA, and ffERG. There was no correlation between BML and ocular findings in agreement with previous similar studies [7, 34, 35]. It is also reported that findings from one eye cannot be similar to the fellow one, so it is highly recommended to evaluate both eyes separately, as we did.

Color vision and CS impairment at high spatial frequencies also were found, being the most frequently observed color vision alteration in the blue-yellow axis. It is well known that the results of the CS measurement are very unspecific, although they are very sensitive. These findings are in agreement with previous studies [33, 36–38].

The most prevalent VF defect pattern was concentric constriction (17 eyes, 29.3%), in agreement with previous studies [39, 40]. This visual impairment may have a central origin (calcarine cortex), as it has been reported previously [41]. In addition, the increased implicit time of P100 in the affected patients, especially in those of Group 1, indicates delayed nerve conduction and involvement of the visual pathway. Group 1 was constituted by a subgroup of patients with evident visual disturbances in their VF test, therefore patients with most severe visual alterations. Consequently, Group 1 showed lower amplitudes and lengthened latencies in PRVER than all patients together as expected. In 2008 da Costa et al. had already reported this finding These results were consistent with the findings obtained in previous publications (27, 39). But data of current series demonstrated a significant retinal involvement showing retinal dysfunction in the ffERG, PERG, and mfERG tests, with both a generalized retinal response loss and a clear alteration of the central retinal area, which could have influenced the results obtained in the VF.

The ffERG showed changes in SRR (the scotopic responses), suggesting that rod cells were impaired in the mercury-vapor intoxicated patients, as well as in OP, suggesting additional involvement of the inner retina.

Besides, results in photopic ffERG responses and the lower amplitude of P50 in PERG found in Group 1, suggest that cones and ganglion macular cells can also be affected in mercury intoxication. These findings harmonize with the psychophysical color-vision losses reported here and in other previous studies and with spatial and temporal luminance contrast-sensitivity losses (16).

Moreover, it is known that ffERG could not be a useful tool for detecting small retinal lesions [42, 43], but the mfERG does and its results add further evidence of damage to the photoreceptors since the amplitudes of the P1 wave showed loss of the retinal response within the central 50 degrees, as reported previously [16]. All these findings reinforce the idea that both the outer and inner retina visual processes are both involved in visual mercury toxicity.

A discrepancy was observed between the dysfunction patterns observed in the VF and the mfERG, with less involvement in the electrophysiological test. This finding also would confirm a visual pathway damage (detected by the mfERG) in addition to that in the retina. Besides, although the PRPEV measurements do not correlate with the BML, patients in group 1 had latencies significantly over 100 milliseconds and significantly reduced P100 amplitudes. Though these results typically occur in optic neuropathies and visual cortex abnormalities, they also can be associated with maculopathies, especially when they are interpreted in conjunction with other retinal function tests (PERG, mfERG, and ffERG). Findings in PRPVE are in agreement with those reported by Ventura et al. and da Costa et al. [16, 27].

Despite the functional retinal involvement and in contrast to the results obtained by Ekinci et al. [6, 15], OCT did not reveal structural changes in the RNFL, macular CRT, and choroid thickness [25, 26]. These differences might be related to the intensity and the manner of poisoning, as the current patients reached higher levels of mercury in a short period of time compared to the long exposure times of workers examined by Ekinci et al. [6, 15].

Current study has several limitations. There were no environmental measurements of mercury either before the accident or during the occupational event. In addition, probably only the most affected patients were evaluated at the IOBA-Eye Institute, and the time that elapsed after the acute accident and the assessment likely was not the most appropriate for adequate follow-up over time. Most of the identified visual alterations seem attributable to the occupational exposure to mercury vapor, but we have not objective information on the ophthalmologic status before the accident. In addition, because a programmed follow-up was not possible, we had no information about the current clinical situation or about the evolution of most patients. Regarding the electrophysiologic tests, most of the patients were minimally affected and the number of patients with significant ophthalmological signs (group 1) was small, thus caution should be taken when interpreting these findings. Finally, the OCT technology has evolved so rapidly that it is possible that with new OCTs based on swept source or ultra-high resolution it would have been possible to detect changes in the retinal or choroidal structures.

Even so, this study presents some relevant findings from a very rare and extremely serious event, for which references are scarce. There was no correlation between BML and ophthalmologic examination findings. As mentioned before, the VA in those patients is slightly affected and there is more VF involvement. The most prevalent VF alteration was diffuse decreased sensibility, but central involvement also was found. This finding could be of retinal and/or neurologic origin considering the mfERG results.

In summary, despite its limitations, this series of patients affected by the same event contributes to the information obtained about mercury poisoning for future similar situations and reinforces the idea of ​​a retinal alteration in addition to CNS damage.

## Conclusions

This is one of the largest series of mercury poisoning reported in the last years in which patients could be analyzed with new ophthalmic diagnostic techniques. Even more it is the only one reporting data on OCT or mfERG after acute exposure to high concentrations of mercury. Finally, findings in the mfERG allowed us to demonstrate that visual impairment after acute events is not only due to neurologic damage, but also to retinal damage at least in those patients with severe lesions on visual field.

## Data Availability

All data and materials are available upon request.
